# Co-circulation of Dengue and Chikungunya Viruses, Al Hudaydah, Yemen, 2012

**DOI:** 10.3201/eid2008.131615

**Published:** 2014-08

**Authors:** Giovanni Rezza, Gamal El-Sawaf, Giovanni Faggioni, Fenicia Vescio, Ranya Al Ameri, Riccardo De Santis, Ghada Helaly, Alice Pomponi, Dalia Metwally, Massimo Fantini, Hussein Qadi, Massimo Ciccozzi, Florigio Lista

**Affiliations:** Istituto Superiore di Sanità, Rome, Italy (G. Rezza, F. Vescio, M. Ciccozzi);; Alexandria University, Alexandria, Egypt (G. El-Sawaf, G. Helaly, D. Metwally);; Army Medical and Veterinary Research Center, Rome (G. Faggioni, R. De Santis, A. Pomponi, F. Lista);; University of Sana’a, Yemen (R. Al Ameri, H. Qadi);; University of Rome Tor Vergata, Rome (M. Fantini)

**Keywords:** Dengue virus, chikungunya virus, viruses, dengue-like illness, Yemen, co-circulation, dengue type 2, mosquitoborne, mosquito-borne, Aedes aegypti, arbovirus infections

## Abstract

We investigated 400 cases of dengue-like illness in persons hospitalized during an outbreak in Al Hudaydah, Yemen, in 2012. Overall, 116 dengue and 49 chikungunya cases were diagnosed. Dengue virus type 2 was the predominant serotype. The co-circulation of these viruses indicates that mosquitoborne infections represent a public health threat in Yemen.

Vectorborne infections are not uncommon in the Middle East (*1*). In particular, recurrent outbreaks of dengue fever have been reported on the Arabian Peninsula since 1990 (*2*). In Yemen, dengue virus (DENV) infections have reemerged with higher frequency during the last decade (*3*); in 2010, during a dengue outbreak that occurred in the southern governorate of Hadramout (*4*), cases of dengue hemorrhagic fever were identified (*5*). In 2010–2011, another mosquitoborne virus, the chikungunya virus (CHIKV), was detected in febrile patients in Al Hudaydah, Yemen (*6*). To evaluate to what extent these arboviruses are involved in dengue-like illness outbreaks in Yemen, we conducted a study in Al Hudaydah.

## The Study

**Figure 1:**
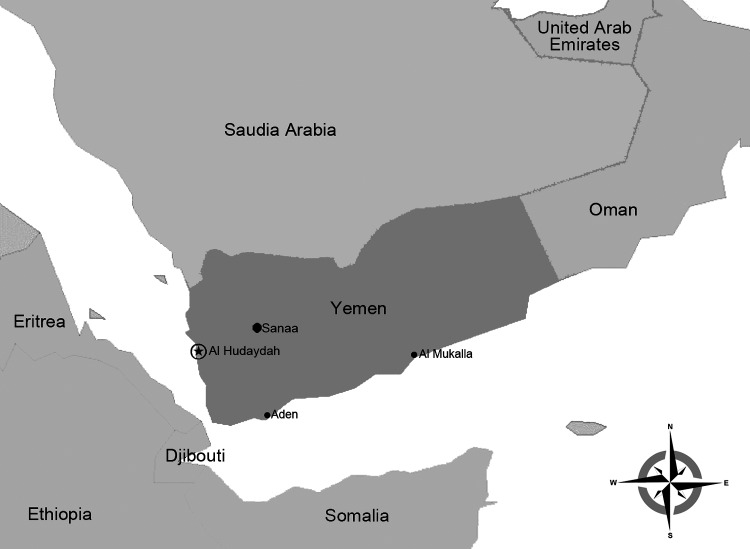
Location of Al Hudaydah, Yemen, where the co-circulation of dengue virus, chikungunya virus, and other dengue-like viruses was studied in 2012. Other important towns, Sanaa, Aden, and Al Mukalla (the capital of Hadramout governorate), are also shown.

The study site was represented by 5 hospital centers (Renal Center, Maritime College, Al Rasheed, Al-Thawra, Al Salakhana) located in Al Hudaydah (Figure 1). Patients hospitalized during 2012 were recruited for the study if they had fever (>37.5°C) and at >2 of the following signs or symptoms at the time of admission: headache, joint pain, muscle pain, skin rash. Serum samples were collected within 4 days from the date of hospital admission and stored and shipped at −20°C.

We used the Maxwell 16 Viral Total Nucleic Acid Purification Kit with the Maxwell 16 instrument (Promega, Madison, WI, USA) according to the manufacturer's instructions to extract nucleic acids from the serum samples. We then analyzed the nucleic acids by using DENV- and CHIKV-specific PCR sequences, as previously described for DENV and adapted for CHIKV (*7*,*8*). To further confirm the results, we amplified and then sequenced *NS1* and *E1* genes from DENV- and/or CHIKV-positive serum samples; the sequences were deposited in GenBank (accession nos. KJ742803–19). 

We used the NovaLisa Dengue IgM-­ and IgG-ELISAs and the NovaLisa Chikungunya IgM- and IgG-capture ELISAs (NovaTec lmmundiagnostica GmbH, Dietzenbach, Germany) according to the manufacturer’s instructions to analyze serum samples for IgM and IgG. Study participants with IgM ELISA– and/or PCR-positive results were defined as recently or acutely infected with DENV or CHIKV.

Overall, 400 persons were enrolled in the study. The median age was 30 years (range 1–60). The median interval between fever onset and sample collection was 4 days (range 2–9).

Of the 400 study participants, 116 (29%) were IgM or PCR positive for DENV RNA. Of those 116 persons, 61 (53%) had IgM, 44 (38%) were positive by PCR, and 11 (9%) had IgM- and PCR-positive results. Of the 55 PCR-positive samples, 41 were DENV-2 and 2 DENV-1. The remaining 12 samples were not typed because the virus titer was low, and it was not possible to achieve a positive signal by using a DENV serotype–specific PCR. Of the 400 study participants, 290 (72.5%) had IgG against DENV. Their distribution by diagnostic category is shown in Table 1.

**Table 1:** Dengue virus–positive study participants by diagnostic category, Al Hudaydah, Yemen, 2012*

Study participants	No. (%) participants by diagnostic category, infection history					
	IgM+/PCR−, recent infection	PCR+/IgM−, acute infection	IgM+/PCR+, acute infection	IgM+ and/or PCR+, acute or recent infection	IgM−/PCR−, no acute or recent infection	Total
Total†	61 (15.2)	44 (11.0)	11 (2.7)	116 (29.0)	284 (71.0)	400 (100.0)
Positive for dengue virus IgG‡	48 (78.7)	22 (50.0)	6 (54.5)	76 (65.5)	214 (75.3)	290 (72.5)

*+, positive; −, negative. †The denominator is represented by the total study population. ‡The denominator is represented by the number of individuals in each category.

Of the 400 participants, 49 (12%) were IgM and/or PCR positive for CHIKV RNA: 38 (77%) were IgM positive, 10 (20%) were PCR positive, and 1 was positive by both methods. Of the 351 patients with negative IgM/PCR results, 33 (9.4%) had IgG against CHIKV.

No samples were PCR positive for both viruses. However, 13 samples had IgM against both viruses, and 1 had positive results for DENV by PCR and CHIKV IgM by ELISA.

**Figure 2:**
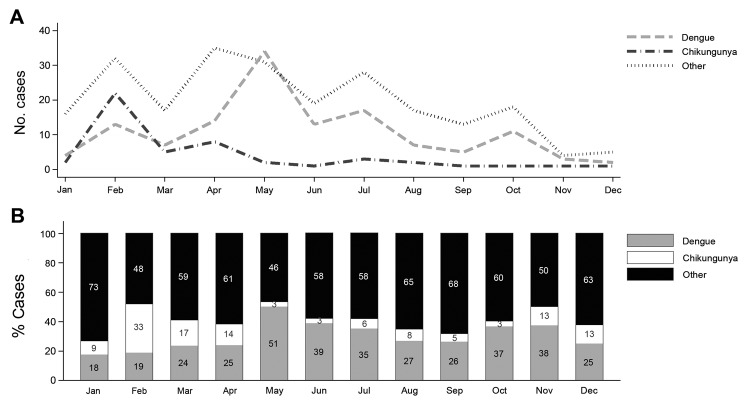
Trends for cases of dengue virus, chikungunya virus, and other dengue-like viruses, Al Hudaydah, Yemen, 2012. A) Number of cases by month. B) Monthly percentages of cases by virus type.

The monthly distribution and the proportion of DENV- and CHIKV-positive cases are shown in Figure 2. Peaks were observed during February, when the highest number of chikungunya cases was observed, and especially May, when the highest number of dengue fever cases was observed. A low number of cases were reported in November and December.

Study participants with CHIKV infection (mean age 30.67 years, + SD 14.17 years) were slightly younger than with DENV infection (mean age 27.53 years, + standard deviation 17.34 years), but the median age (30 years) and range (1–60 years) were the same for the 2 groups. The distribution of signs and symptoms for recent/acute cases of dengue and chikungunya are shown in Table 2. The most common signs symptoms for both infections were joint pain (98%), myalgia (95% and 94%, respectively), and headache (94% and 88%, respectively). Persons with chikungunya were more likely than those with dengue to report vomiting (41% vs. 25%). No other major difference in the frequency of specific signs or symptoms was found.

**Table 2:** Clinical characteristics of study participants with dengue and chikungunya, Al Hudaydah, Yemen, 2012

Sign or symptom	Participants with signs or symptoms of dengue, n = 116			Participants with signs or symptoms of chikungunya, n = 49		Difference*	SE	P value
	No. (%)	SE		No. (%)	SE			
Fever	116 (100)	0		49 (100)	0	0		
Arthralgia	114 (98)	0.01		48 (98)	0.02	0	0.02	0.890
Myalgia	110 (95)	0.02		46 (94)	0.03	0.01	0.04	0.807
Headache	109 (94)	0.02		43 (88)	0.05	0.06	0.05	0.178
Abdominal pain	93 (80)	0.04		35 (71)	0.07	0.09	0.07	0.221
Backbone pain	85 (73)	0.04		32 (65)	0.07	0.08	0.08	0.306
Watery diarrhea	56 (48)	0.05		27 (55)	0.07	−0.07	0.09	0.426
Rash	35 (30)	0.04		13 (27)	0.06	0.04	0.08	0.640
Vomiting	29 (25)	0.04		20 (41)	0.07	−0.16	0.08	0.042

*Difference between proportions.

## Conclusions

Our data provide evidence of co-circulation of CHIKV and 2 DENV serotypes in the governorate of Al Hudaydah. DENV-2 was the predominant serotype in our study population. Whether this serotype was newly introduced into Yemen in 2012 is unknown. In 2011 in the same area, a small study was conducted with 47 patients with dengue-like illness, and only 3 were PCR positive for DENV: 2 for DENV-1 and 1 for DENV-3 (data not shown). In 2010 in Al Mukalla, the capital of the district of Hadramout, DENV-3 was also detected in patients with dengue fever and dengue hemorrhagic fever (*5*,*9*).

The high prevalence (>70%) of DENV IgG in our study population suggests past exposure to DENV. Relatively high rates of IgG were also found during the outbreak that occurred in 2011 in Hadramout, where 28% of DENV IgM–positive and 43% of DENV IgM–negative patients with dengue-like illness were positive for DENV IgG (*4*). The identification of dengue hemorrhagic fever cases caused by DENV-3 in Al Mukalla is also suggestive of exposure to different DENV serotypes (*5*). The hypothesis of continued reintroduction of different DENV serotypes in Yemen dates back to 1983, when a secondary heterotypic DENV infection was suspected in a man who had traveled from Dalah (160 miles from Aden), an area endemic for DENV. The traveler had dengue hemorrhagic fever and high antibody titers against all 4 DENV serotypes (*10*). It is well known that travels to and from DENV-endemic areas are common, and imported cases from eastern Africa (Zanzibar) were reported in Yemen as early as the nineteenth century (*11*,*12*). Circulation of different DENV serotypes has also been reported in Saudi Arabia: DENV-3 emerged as the predominant serotype after 1997, and DENV-2 and DENV-1 serotypes had been associated with earlier outbreaks (*13*).

We identified a rather large number of cases of recent or acute CHIKV infection. This finding is consistent with a previous estimate of 1,542 cases during October 2010–January 2011 (*6*) and with the detection of CHIKV RNA in *Aedes aegypti* mosquitoes, the dominant type of mosquito in entomologic investigations conducted in Al Hudaydah (*14*).

The circulation of mosquitoborne viruses in a dry area like Yemen is not surprising. Water scarcity and lack of infrastructure in periurban areas require regular storage of water for household and potable use, and water containers favor *A. aegypti* mosquito reproduction. Moreover, increasing migration and urbanization may favor the introduction and spread of these infections (*15*).

Our study did have some limitations. First, the study was hospital based. Thus, the findings may not be representative of the whole epidemic in the community. Second, some misclassification of the cases caused by the sensitivity and specificity of the tests or to a time lag between symptom onset and sample collection (i.e., PCR- or IgM-negative results caused by testing intervals that were too long or too short, respectively) cannot be ruled out. Last, the prevalence of IgG against DENV might have been overestimated because of possible cross-reactivity with other flavivirus infections.

In conclusion, CHIKV and various DENV serotypes co-circulate in Yemen’s port city of Al Hudaydah. The detection of CHIKV and DENV IgG–positive persons suggests that these viruses are either endemic or continuously reintroduced to the area. Mosquito control activities are needed to reduce the effect of arbovirus infections on public health.
